# Giant ascending aortic aneurysm: A rare case report

**DOI:** 10.1016/j.radcr.2025.02.030

**Published:** 2025-03-18

**Authors:** Thirafi Mitsali, Dian Komala Dewi, Hilman

**Affiliations:** Faculty of Medicine University of Padjadjaran, Dr. Hasan Sadikin General Hospital, Department of Radiology, Pasteur No.38, Bandung City, West Java 40161, Indonesia

**Keywords:** Giant aortic aneurysm, Ascending, Atherosclerosis

## Abstract

A giant ascending aortic aneurysm (AscAA), defined as an aneurysm larger than 10 cm, is a rare and potentially life-threatening condition that often remains asymptomatic until it reaches a critical size. Atherosclerosis is the most common cause in elderly patients, and imaging plays a crucial role in diagnosis and management. In this case, a 72-year-old man presented with intermittent sharp chest pain radiating to the back, progressive hoarseness over 5 years, and shortness of breath. Imaging revealed a 12.51 cm × 11.27 cm × 10.0 cm saccular aneurysm with calcified plaques, consistent with a Stanford Type A aortic aneurysm and underlying atherosclerosis. Surgical intervention remains the only definitive treatment, although it carries significant risks. Early diagnosis, timely surgical intervention, and postoperative surveillance are critical in improving patient outcomes for this high-risk condition.

## Introduction

Giant ascending aortic aneurysm (AscAA) are rare and often progress without symptoms in their early stages. However, as they enlarge, the risk of life-threatening complications such as dissection or rupture increases significantly, particularly when the diameter surpasses 6 cm [1]. In older adults, arteriosclerotic degeneration is the primary cause of AscAA, while in younger individuals, conditions like Marfan syndrome or bicuspid aortic valve are more common etiologies. Enlarged aneurysms may compress or erode adjacent structures, leading to symptoms such as chest or back pain, or hoarseness due to recurrent laryngeal nerve involvement [2].

Diagnostic evaluation typically involves imaging modalities like chest radiography, echocardiography, contrast-enhanced computed tomography (CT), and magnetic resonance angiography, with contrast-enhanced CT being particularly effective for detailed assessment of aneurysm morphology. While conservative management often results in poor outcomes, surgical intervention remains the definitive treatment to improve survival, despite the associated procedural risks, especially when combined with aortic valve or arch replacement [3].

## Case presentation

A 72-year-old man was admitted to our department presenting with intermittent chest pain, which he had experienced for the past 4 years and had worsened over the last 6 months. The chest pain is sharp and penetrating, radiating to the back, and accompanied by shortness of breath. Additionally, the patient reported progressive hoarseness of voice over the past 5 years, which had previously led him to seek treatment from an ENT specialist. The patient reported no symptoms of cough, fever, night sweats, weight loss, fatigue, lumps on the body, bowel or urinary disturbances, or swelling in both legs.

The patient had no prior similar symptoms. He denied a history of asthma, tuberculosis or TB contact, heart disease, diabetes mellitus, autoimmune disease, or any malignancy in the family. However, he had a history of hypertension for which he routinely took medication. He is a smoker with a 20-year history of smoking 1 pack per day. In terms of treatment history, the patient has not undergone any surgeries or treatments such as chemotherapy or radiotherapy.

**Fig. 1:**
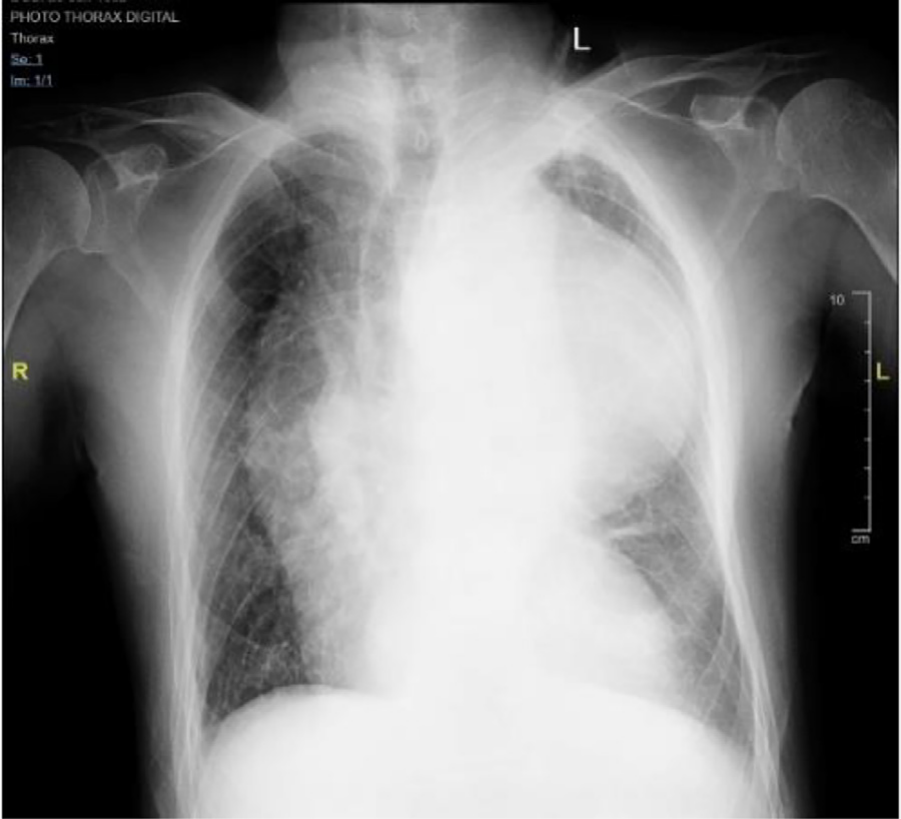
Aneurysm appearance on chest X-ray examination.

Chest X-ray examination (Fig. 1) revealed cardiomegaly with a cardiothoracic ratio (CTR) of 60.2%. Left hilar opacity and paratracheal widening were observed, with no pleural effusion or significant lung pathology. Additionally, there was evidence of calcification in the aortic arch and ascending aorta, indicating atherosclerotic changes.

Nongated CCTA using a 256-slice CT scanner revealed a calcium score of 609, with notable values for the LAD (255) and RCA (354). The LM and LCx arteries were free of calcifications or stenosis, while the LAD and RCA showed moderate stenosis due to calcified plaques. Cardiac structures were normal, including atrial and ventricular dimensions and a trileaflet aortic valve. A saccular aneurysm was identified from the ascending to proximal descending aorta, with partially calcified plaques noted. Pulmonary artery dimensions were normal. The findings indicate coronary artery disease (CAD) and a Stanford Type A saccular aortic aneurysm, necessitating comprehensive management and follow-up.

**Fig. 2:**
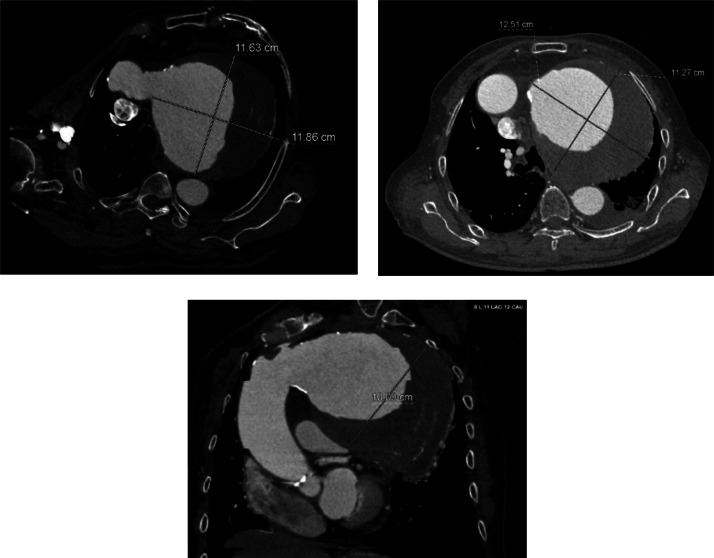
Cardiac computed tomography scan with contrast showing giant ascending aortic aneurysm.

**Fig. 3:**
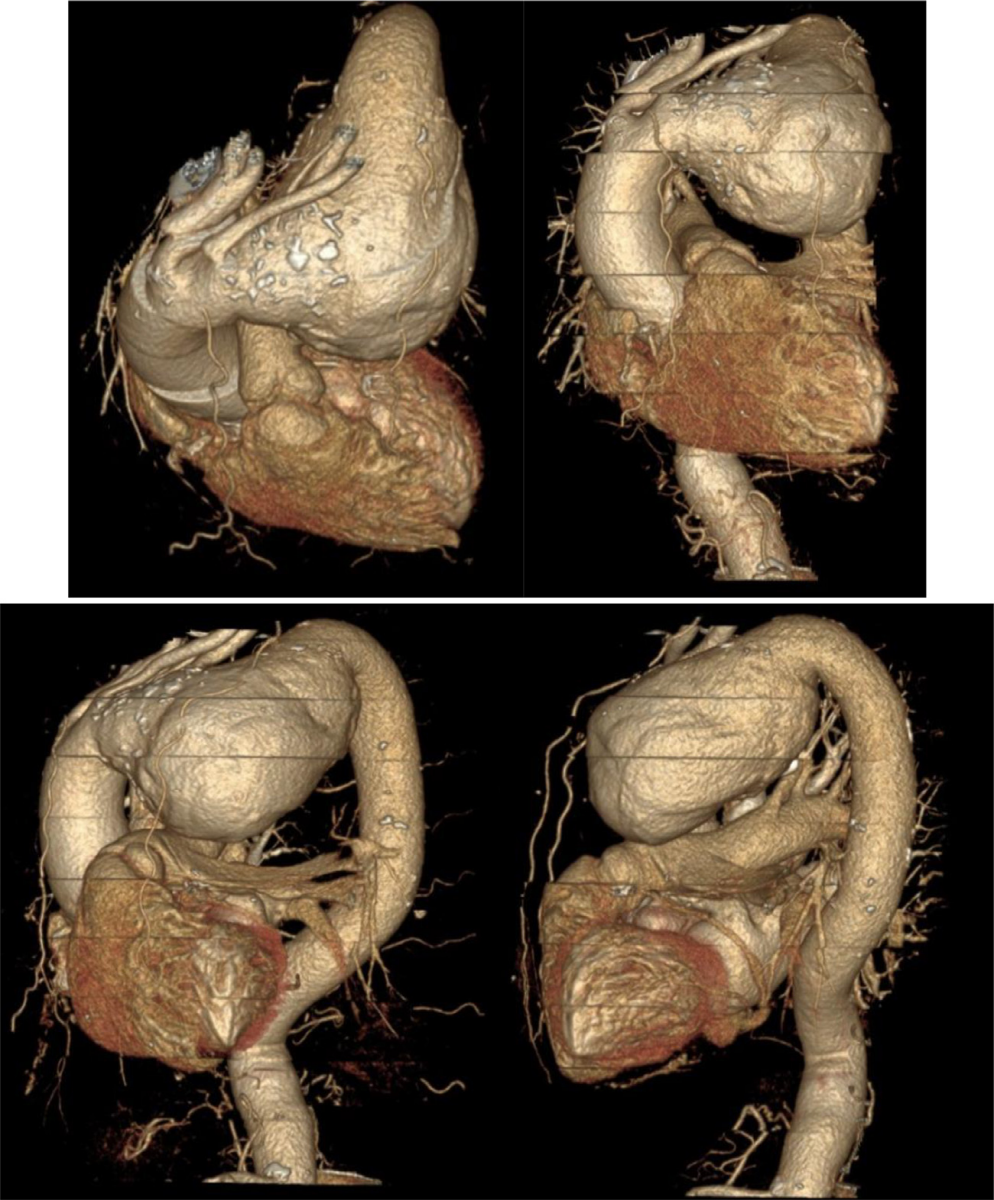
Three-dimensional volume-rendered images of cardiac computed tomography scan showing giant ascending aortic aneurysm.

A contrast-enhanced CT thorax scan demonstrated an enlarged aortic arch with a saccular aneurysm measuring approximately 12.51 cm × 11.27 cm × 10.0 cm, involving the proximal descending aorta. The aortic wall displayed irregular calcifications. The aneurysmal dilation caused mass effect on adjacent structures, but no significant compression of vital mediastinal structures was reported (Fig. 2, Fig. 3).

Further evaluation with Doppler ultrasound showed stable plaques in both femoral arteries and no evidence of deep vein thrombosis or chronic venous insufficiency in the lower extremities. Normal arterial flow was confirmed bilaterally. The findings support the diagnosis of an aortic aneurysm with atherosclerosis and suggest the need for further cardiovascular surgical evaluation.

## Discussion

Giant ascending aortic aneurysm (AscAA) is the most common subtype of thoracic aortic aneurysm (TAA), accounting for approximately 60% of TAA cases and involving the ascending aorta or aortic root. Giant AscAA is defined as an aneurysm with a diameter of more than 10 cm, although this condition is rare [4]. TAA is more common in males than females, with a ratio of 1.7-3:1, and the average age of patients is 65 years at diagnosis [5]. In this case, the patient was 72-year-old male, matching the prevalence found in previous studies.

The aorta is divided into 2 main parts, the thoracic aorta and the abdominal aorta. The thoracic aorta consists of 3 segments: the ascending aorta, the aortic arch, and the descending aorta. The ascending aorta, about 5 cm long, begins at the aortic valve and ends at the brachiocephalic artery. This segment consists of 2 parts, the aortic root which includes the sinus of Valsalva and sinotubular junction (STJ), and the tubular ascending aorta which extends from the STJ to the aortic arch. More than half of TAA is found in the ascending aorta, as in this case, including both the aortic root and its tubular segment [6].

Major risk factors for aortic dilatation and aneurysm include age, gender, height, hypertension, smoking history, and severe atherosclerosis, with age being the most significant predictor for dilatation of the ascending aorta [6]. In this case, the patient had a history of hypertension, but he was taking medication regularly. The patient was also a long-term smoker. In young patients, Marfan syndrome and bicuspid aortic valve are often the triggers. Other etiologic factors include trauma, aortic pseudoaneurysm, aortic dissection, and vasculitis diseases such as Takayasu arteritis and giant cell arteritis [4].

TAA aneurysm formation involves a combination of structural disruption of the aortic wall, dysfunction of molecular signaling pathways, and genetic mutations. Structurally, aging leads to fragmentation of elastic fibers, loss of smooth muscle, and replacement with amorphous material known as cystic medial degeneration. These conditions increase the stiffness and weaken the aortic wall, making it prone to dilatation, especially in the ascending aorta. As per Laplace's law, aortic dilatation increases wall stress, triggers vascular remodeling, and accelerates dilatation further. In addition, disruption of the TGF-B1 signaling pathway, which is essential for maintaining the integrity of the vascular wall, contributes to the damage. Uncontrolled activation of TGF-B1 promotes matrix degradation through the production of plasminogen activator and the release of matrix metalloproteinases. Genetic factors also play an important role, such as ACTA2 mutations that affect smooth muscle actin and MYH11 mutations that alter smooth muscle contractile proteins, increasing the risk of aneurysms. Mutations in fibrillin 1 in Marfan syndrome weaken the vascular wall while disrupting the regulation of TGF-B1 bioavailability. Other disruptions in the TGF-B signaling pathway, such as in Loeys-Dietz syndrome, as well as GLUT10 and SMAD3 mutations, also exacerbate vascular wall damage and favor the development of TAA aneurysms [6].

Most patients with AscAA are asymptomatic and are usually detected incidentally through radiologic imaging examinations. However, larger aneurysms may present with varied clinical symptoms due to compression or erosion of surrounding structures and organs, such as the chest wall, heart chambers, pulmonary arteries, trachea and oesophagus, resulting in pain in the chest or back. Patients may also present with hoarseness due to laryngeal nerve paralysis [4]. In this case, the patient complained of intermittent sharp chest pain that was progressive and radiated to the back, accompanied by shortness of breath and hoarseness, in accordance with previous studies. The patient's complaints were best explained by the findings on the contrast-enhanced thoracic CT scan, which showed that the aneurysm dilation caused a mass effect on nearby structures, although there was no significant compression of vital mediastinal structures.

Optimized imaging of the aortic anatomy and pathology, especially in cases of giant thoracic aortic aneurysm, requires the use of dedicated aortic imaging protocols. Several imaging modalities, including computed tomography (CT), magnetic resonance imaging (MRI), transthoracic echocardiography (TTE), transesophageal echocardiography (TEE), and abdominal aortic ultrasound, play crucial roles in these evaluations. The selection of the appropriate imaging technique depends on patient-specific factors such as hemodynamic stability, contrast allergy, renal function, and tolerance to the procedure, with MRI sometimes requiring sedation due to longer examination times and its confined space. The availability of imaging modality at the institution and the expertise of the imaging physician also guides the decision. Among these methods, CT has become the most widely adopted due to its rapid acquisition of high-resolution, three-dimensional (3D) imaging data, which has largely replaced diagnostic catheter angiography for assessing aortic pathology and guiding periprocedural vascular evaluations [7]. The imaging modalities used in this case is a nongated cardiac CT angiography followed with a contrast-enhanced CT scan of the thorax, after previously an aneurysm was found on initial thorax X-ray. Additionally, a Doppler ultrasound was performed to detect plaque in the femoral artery, as well as to assess for deep vein thrombosis (DVT) or chronic venous insufficiency (CVI), however no DVT or CVI was found.

Conservative management of AscAA results in a low life expectancy. Surgical intervention is the only effective treatment option. However, surgical repair of AscAA carries a high risk of mortality, particularly when it is combined with aortic valve replacement or concurrent arch replacement [8].

Current guidelines for the management of TAA recommend elective surgery when the risk of adverse events, such as dissection, rupture, or sudden death, outweighs the surgical risks. Aneurysms with a maximum diameter of 5.5 cm are generally considered the threshold for elective surgical intervention, based on natural history studies that link larger diameters with increased risk of rupture or dissection. Although growth rates of ascending aortic aneurysms are often slow, rapid growth of 0.5 cm in a year or sustained growth of 0.3 cm over 2 consecutive years remains an indication for surgery. Additionally, aneurysms with a diameter of 5.0-5.4 cm may also warrant surgery, particularly if the patient's surgical risk is low. For patients undergoing aortic valve surgery with concurrent aneurysm of ≥4.5 cm, guidelines suggest performing simultaneous aortic replacement, especially in those with bicuspid aortic valve (BAV). However, in patients without aortic aneurysm surgery, aneurysms tend to grow slowly with low complication rates. Overall, the decision to operate should be individualized, considering factors such as the patient's age, comorbidities, and the type of aortic valve surgery, with prophylactic aortic replacement considered for those with aneurysms ≥5.0 cm [7].

Surgical management of giant AscAA is challenging due to the significant risks of complications such as bleeding, organ ischemia, underlying diseases, and the need for staged repairs. During the operation, performing a median sternotomy can pose a risk of aortic injury, as the aneurysmal wall is very close to the sternum. Exploration and cannulation of the femoral vessels for cardiopulmonary bypass before performing the median sternotomy is recommended to mitigate the risk. Additionally, protecting neurocerebral functions through antegrade and retrograde cerebral perfusion is crucial to prevent neurological complications. Deep hypothermia and circulatory arrest may also be preferred during surgery to further safeguard the patient's wellbeing [4].

In patients with TAA and elevated blood pressure (systolic BP ≥130 mm Hg or diastolic BP ≥80 mm Hg), antihypertensive medications are recommended to reduce cardiovascular risks. Beta blockers are commonly used for controlling blood pressure, in absence of contraindications, and angiotensin II receptor blockers (ARBs) can be used as adjunct therapy. The main goal of blood pressure management in TAA is to slow aneurysm growth, prevent aortic dissection, and reduce the risk of other cardiovascular issues, such as heart attack and stroke. Achieving a target systolic BP of ≤130 mm Hg and diastolic BP of ≤80 mm Hg is important for reducing the risk of complications. While there is limited data, aiming for a more stringent systolic BP target of <120 mm Hg could be beneficial for certain patients who are not undergoing surgery [7].

The primary concern in the management TAA is the complications, notably the risk of rupture or dissection. The likelihood of rupture is increasing significantly when the aneurysm diameter exceeds 6 cm. In addition to size, rapid expansion and calcification of the aneurysm are also associated with a higher rupture risk [8].

After TAA repair, surveillance imaging is critical for detecting complications or monitoring any progression of aortic pathology. CT is the preferred modality for this purpose, although MRI is a viable alternative despite limitations like metallic artifacts. Open repair of the thoracic aorta has proven durable with low rates of reintervention (1%-7% over 10 years). In contrast, Thoracic Endovascular Aortic Repair (TEVAR) is associated with a higher rate of complications such as endoleaks, dissection, and stent-graft issues, as well as a reintervention rate of 7%-23%. Surveillance after TEVAR can reveal complications like endoleak or an increase in aortic size. While MRI avoids ionizing radiation, it is more expensive, time-consuming, and has lower resolution compared to CT. Typically, if no complications are found after open repair, surveillance intervals are extended, while annual imaging is recommended for patients with abnormal findings or those requiring reintervention [7].

## Conclusion

Treating large ascending aortic aneurysms is still a difficult task and has been linked to high death rates in the past. However, improvements in surgical methods—like better graft materials, improved heart and brain protection, transesophageal echocardiography, and better blood management—have greatly improved patient outcomes. Referring patients early, performing surgery promptly, and providing careful care after surgery are key to reducing complications and increasing survival in these high-risk cases.

## Patient consent

Written informed consent for publication of their case was obtained from our patient.
